# Mental health service preferences in rural Australia: the importance of culture and connection

**DOI:** 10.1080/00049530.2025.2544924

**Published:** 2025-08-18

**Authors:** Margot P. Moody, Natasha M. Loi, Adam J. Rock, Kim J. Usher, Kylie Rice

**Affiliations:** a School of Psychology, University of New England, Armidale, Australia; b School of Health, University of New England, Armidale, Australia

**Keywords:** Rural, mental health, accessibility, telehealth, preferences

## Abstract

**Objective:**

There is a lack of adequate mental health services available in rural and remote Australia, with rural Australians experiencing poorer mental health outcomes than those in urban areas. Service access needs to improve, and the current study aimed to address this by exploring the acceptability of services, including telehealth, among rural Australians.

**Method:**

A convergent mixed-method online survey was used to examine mental health service and clinician preferences via a series of open-ended and scaled questions. A total of 294 rural and regional Australians participated in the study, and textual responses were analysed using reflexive content analysis, with a repeated measures analysis of variance utilised to further examine telehealth acceptability.

**Results:**

Results indicated a preference for in-person support that was accessible and available, with clinicians who were qualified and clinically competent. The importance of cultural competence was also highlighted, with responses indicating a need for clinicians with both rural and local knowledge, who also fostered connection and trust with their clients. Likewise, participants demonstrated an increasing acceptability of telehealth, provided the clinician was rurally based.

**Conclusions:**

The results indicate a need for mental health services in rural Australia to be acceptable and relevant in order to best meet the needs of this population. Implications for future service delivery in rural areas, including recommendations for further research, are discussed.

## KEY POINTS

**What is already known about this topic:**

1. Mental health service availability is a barrier for rural Australians seeking mental health support, with fewer psychologists working in rural communities compared to urban communities.
2. Telehealth services have been utilised to increase service availability in rural areas. However, emerging evidence suggests a preference for in-person support amongst rural people.
3. It is important for mental health clinicians to be culturally responsive, particularly when working in rural communities.

**What this topic adds:**

1. Results add to the literature regarding rural people’s preference for in-person support, though suggest an increasing acceptability for telehealth should the providing clinician be based rurally.
2. Rural people want mental health clinicians who have both local and rural knowledge of their communities and focus on building connection and trust with their clients.
3. Though service availability needs to increase in rural communities, such supports also need to be acceptable and relevant to rural people, to ensure uptake of services.

Rural Australians are recognised to face greater barriers to mental health service access compared to urban Australians, leading to poorer mental health outcomes (Matta et al., 2021). Limited service availability remains a significant barrier to service access (Kavanagh et al., 2023). For example, in 2021, there were 142 psychologists per 100,000 people in major cities and just 47 psychologists per 100,000 people in remote areas (Australian Institute of Health and Welfare [AIHW], 2021). While rural people seek and access similar rates of mental health support as urban people (Batterham et al., 2020; Fitzpatrick et al., 2021), there are substantial differences in the type of support they receive. Rural people are more likely to receive support from a general practitioner than a psychologist (Batterham et al., 2020; Dolja-Gore et al., 2014), with an increasing use of medication, rather than psychological therapies, being used to treat mental health concerns (Fitzpatrick et al., 2021).

Services available to rural Australians also need to be culturally responsive; that is, contextualising a person’s own beliefs and attitudes within both their cultural knowledge and generalist and specialist skill set (Mullins & Khawaja, 2018). It is particularly important for clinicians and services in rural communities to be culturally responsive due to the higher proportion of Indigenous peoples in such communities (AIHW, 2022). Clinicians and service providers also need to be mindful of the unique culture in rural communities tied with people’s rural identity, including traits such as stoicism and self-reliance (Cheesmond et al., 2019). This identity impacts the way rural communities want their services delivered, with rural people indicating a preference for rural-specific support, including clinician knowledge regarding rural issues (Gunn et al., 2013). Clinicians need to be mindful not only of rural culture broadly but also acknowledge that each rural community has its own local culture and practices (Bischoff et al., 2014).

Indeed, person-centred and collaborative care has been found to be a key facilitator in improving service access in rural communities (Kavanagh et al., 2023). Numerous researchers have suggested that the need for service delivery should be informed by rural communities’ wants and needs (Bischoff et al., 2014; Boyd et al., 2007; Pelling & Butler, 2015), arguing that mental health disparities in rural areas should be addressed through both the availability and acceptability of healthcare (Bischoff et al., 2014). Though service availability remains a significant issue for rural communities (Kavanagh et al., 2023; Moody et al., 2025, in review), consideration must also be made for the acceptability of services. That is, the degree to which a person perceives a service as acceptable in meeting their needs can impact their ability to engage with such a service (Bischoff et al., 2014).

In rural areas, literature and policymakers have looked towards the use of telehealth and other e-mental health services (Bradford et al., 2016) in order to improve the availability of services. While telehealth often refers to a range of health services delivered via technology (such as video or phone); telehealth can, more broadly, be defined as any service between a client and medical practitioner performed as an alternative to in-person consultations (Australian Health Practitioner Regulation Agency, 2023). Since the start of the COVID-19 pandemic which led to greater acceptance of such services, telehealth usage has increased (Amos et al., 2022; Jayawardana & Gannon, 2021). However, telehealth is not without its challenges, with users citing issues with privacy, connectivity, and difficulty developing rapport (Fisher et al., 2024). Due to the resources, it requires, telehealth is less accessible to those in socioeconomically disadvantaged households (Alam et al., 2019). Fly-in-fly-out services have also been used to replace consistent in-person support, this work can be challenging for clinicians (Hussain et al., 2015). Such services may also be less appropriate for Indigenous and remote communities, given the care that needs to be taken to develop long-term relationships and credibility in communities that are often used to inconsistent services (Sutherland et al., 2017).

Though the use of telehealth is a necessary strategy to improve the availability of mental health services in rural areas, less attention has been paid to the acceptability of such services. Across Australia, the acceptability of telehealth compared to traditional face-to-face services is low (March et al., 2018), with some telehealth modalities indicating low commencement and completion rates amongst users (Waller & Gilbody, 2009). For some rural and remote communities where the only access to mental health services is via telehealth, reluctance to use this modality may mean people go without support. Though this reluctance could be construed as an attitudinal barrier, previous research by the authors found that rural Australians demonstrated more positive attitudes towards seeking mental health support, including greater mental health literacy and lower levels of personal stigma, than urban Australians (Moody et al., 2025, in Review). Rather, findings suggested that service barriers were the most prominent issue for rural communities when seeking support.

In order to better understand such service barriers, a thorough literature review regarding mental health service preferences and telehealth acceptability in rural Australia was conducted with limited research evident on this topic. For example, Amos et al. (2022) reported that remote communities strongly preferred in-person support over telehealth. Likewise, a systematic review suggested that rural/remote youth also preferred in-person support, indicating a preference for telehealth only as an adjunct, rather than a replacement, to face-to-face services (Mseke et al., 2023). Given the necessary reliance on telehealth to deliver services in rural communities (Amos et al., 2022), a greater insight into this population’s preferred mental health service modalities is warranted. This information would allow for services such as telehealth to be tailored to rural communities, ensuring not only the availability but also the acceptability and, therefore, uptake of such services. The current study aimed to address this gap in the literature by understanding service and clinician acceptability, through both service modalities preferences and the desired skills and qualities of clinicians. An additional aim was to explore the acceptability of telehealth and whether attitudes towards telehealth differed depending on the rural or urban background of the providing clinician.

## Method

### Participants

A total of 527 Australian adults participated in a survey related to a broader research project, including 372 via an online data collection agency (Online Research Unit; ORU), and 155 via a community sample. As the current study focused on regional and rural responses only, 219 participants were not included in the final sample due to being from an urban location. A further 14 participants were removed due to incomplete data across responses. Due to the survey containing multiple short-answer questions, the decision was made not to remove participants with partial or incomplete responses; rather, the number of participants who provided a response is included for each question discussed, with the number of responses represented as *n*. The final sample included 294 participants, with 195 (66%) from regional areas and 99 (34%) from rural areas. See Table 1 for the sample demographics.

**Table 1.** Participant demographics (*N* = 294).

Location	Regional		Rural	
	195 (66%)		99 (34%)	
Age in years (*M*)	57.9		53.9	
	*n*	%	*n*	%
Gender				
Female	113	57.7	72	72.7
Male	80	40.8	25	25.3
Non-binary/third gender	1	0.5	2	2
Prefer not to say	2	1	2	2
Aboriginal/Torres Strait Islander status				
Does not identify	193	98.5	91	91.9
Aboriginal/Torres Strait Islander	3	1.5	8	8.1
Employment status				
Working full time	31	15.8	27	27.3
Working part time	42	21.4	17	17.2
Unemployed	9	4.6	8	8.1
Homemaker/Stay at home parent	14	7.1	7	7.1
Student	3	1.5	3	3
Retired	75	38.3	22	22.2
Other	22	11.2	15	15.2
Education				
Primary	1	0.5	–	–
Secondary	46	23.5	23	23.2
Vocational	51	26	28	28.3
University	72	36.7	37	37.4
Graduate or professional Degree	26	13.3	11	11.1

### Procedure

Participants accessed an online convergent mixed methods survey via Qualtrics^TM^ (Provo, UT). Items included a series of scaled and open-ended questions regarding service and clinician preferences. Ethics approval was provided by the university’s Human Research Ethics Committee (Approval No.: HE23–159). Data collection occurred from February to July 2024. Participation in the survey was voluntary, and adults aged 18 years and over were invited to participate. Recruitment was completed via ORU, social media advertising (e.g., Facebook), and community networks, with those recruited via ORU receiving a notional credit for their participation. Prior to completing the survey, participants read the information sheet and provided implied consent.

### Materials

#### Demographics

Participants provided their age, postcode, gender, employment status, cultural background and education. Rurality was classed using the Australian Statistical Geographical Standard Remoteness Structure (Australian Bureau of Statistics, 2023). Any postcodes that partially fell within the *inner regional* category were classed as *regional* with all remaining postcodes classed as *rural*. Initial analyses indicated similar categories and clinician preferences across both locations; therefore, for the purposes of the current study, responses were combined with all respondents here-on referred to as *rural*.

#### Clinician preferences

Participants were asked to rate their acceptability of a telehealth service based on the clinician’s background, such as a city-based clinician, rural-trained clinician, or a rural-based clinician. Specifically, participants were asked: “*If you were to access mental health support via telehealth, how likely would you consider seeing the following types of clinicians?”* Participants responded on a 5-point Likert scale (1 = *extremely unlikely*; 5 = *extremely likely)*. The 5-point scale was chosen for its suitability for the statistical analyses used for this question, while also allowing participants to provide a neutral response by way of a midpoint (Chyung et al., 2017).

#### Short-answer questions

Participants were asked to respond to a series of open-ended questions relating to their current access to mental health services, preferred clinician skills and qualities, and preferences towards telehealth versus in-person services. For example, participants were asked: *“What are the current mental health services available in your community?”, “If you were to access a mental health service, what type of service would you prefer?/Why?”, “What would you want your clinician to know about your community?”, “What skills and qualities would you want your clinician to have?”, “In what circumstances would you consider using telehealth to see a mental health clinician?”* and *“In what circumstances would you consider seeing a mental health clinician in person?”*

### Statistical methods

#### Quantitative analysis

Quantitative analyses were performed using SPSS version 29. To examine differences in the acceptability of telehealth clinician background, a repeated measures analysis of variance (ANOVA) was conducted with clinician type as the independent variable and consisting of three groups: city-based clinician, rural-trained clinician, and rural-based clinician.

#### Content analysis

Short-answer questions were analysed using reflexive content analysis (RCA), to align with the overall research aim of understanding participants’ perceptions of services. RCA aims to identify meanings true to the dataset and participant experiences, rather than a latent interpretation (Nicmanis, 2024). Coding and analysis were conducted as per O’Connor et al. (2015). Initial coding was completed by the first author and a second coder, with the remaining coding completed by the first author. Codes were reviewed by the second coder as necessary with both coders being researchers and practitioners trained in clinical psychology. The first author created subsequent categories and subcategories, which were then reviewed by the research team. This approach to content analysis was taken to allow for reflexivity in analysis by participating in regular discussions regarding categories and codes and allowing clarification and adjustments as necessary (Nicmanis, 2024; O’Connor et al., 2015).

## Results

### Services available

Participants were asked to identify both services available to them and services they would prefer. Across both questions, respondents identified services relating to *specified in-person support* (henceforth referred to as *in-person support*), *other services*, and *personal support*. *In-person support* encompassed a range of face-to-face services and was differentiated from the other categories to include services typically delivered one-on-one and in-person, as opposed to broader or non-specific services. *Other services* included community-based programs, online supports (such as Lifeline and Beyond Blue), or specialised services such as workplace programs. *Personal supports*, which were unique to the individual, referred to non-mental health services such as social support and self-care. A number of responses indicated no services were currently available to participants (*n* = 31), nor did they have a preference for a service (*n* = 11). Frequency and percentages of all categories are included in Table 2.

**Table 2.** Service availability.

Category	Example codes	Services Available (*N* = *283*)	Services Preferred (*N = 255*)
		*n*	*n*
In-person support		275	208
Mental health	Psychologist, psychiatrist	113	119
Medical	GP, hospital	162	36
Any individual support	One on one	–	53
Other services		184	48
Community supports	Community services/support	87	13
Online supports	Online crisis support, online resources	79	27
Specialised services	Workplace support, group support	19	8
Personal supports	Family/friends, personal wellbeing	18	–
Service qualities	Confidential, free, qualified	–	32
No services		31	11

*Note. n* represents number of responses provided.

*In-person support* included both mental health practitioners and medical services. Though the majority of respondents identified an in-person service currently available to them, when responding to preferred services, responses indicated a greater preference for mental health services than medical services. A number of responses (*n* = 53) also indicated no preference for a specific service or support, as long as it was in person. Though over half of responses identified a service in the *other services* category as currently available, a smaller number (*n* = 48) indicated this was a preferred service. Likewise, *personal supports*, only appeared as an available service, not a preferred service. A category relating to *service quality* was identified amongst the preferred services responses. Instead of identifying specific services, respondents indicated preferences for service qualities and approaches (such as qualified clinicians, confidentiality, or one-on-one support), regardless of the service delivery mode.

### Factors influencing service preference

Participants were asked to provide a rationale for their preferred service, and to also comment on what circumstances they would utilise telehealth or in-person mental health support. Across these questions, four main categories were identified: *if in crisis, accessibility, connection*, and *trusted service*. Regarding the acceptability of services, a number of responses indicated that under no circumstances would participants use telehealth (*n* = 53) or in-person support (*n* = 12), though no reason was provided. Likewise, some responses indicated that they would consider telehealth (*n* = 8) or in-person support (*n* = 51) under any circumstances. See Table 3 for frequency and percentages of categories.

**Table 3.** What do rural people seek in a service?

Category	Example Codes	Reason for Preferred Service (*N* = *233*)	Telehealth Services (*N = 259*)	In-Person Support (*N = 251*)
		*n*	*n*	*n*
If in crisis	If in crisis, only option, in-person/online unavailable	–	154	107
Connection	Connection, continuity of care	103	6	11
Accessibility	Accessible, available	60	67	70
Logistics	Travel, finances	12	30	20
Trusted service		121	23	34
Experience	Positive/negative experience	34	13	1
Competence	Effectiveness, knowledge	67	2	4
Reputation	Trusted service, reputable	20	2	4
Referred	GP referred		6	25
No circumstances		–	53	12
Any circumstances		–	8	51

*Note. n* represents number of responses provided.

#### If in crisis

When considering the acceptability of telehealth or in-person support, nearly half of the respondents indicated the use of either service if they were in crisis, if it was the only option, or if the alternative service was not available.

#### Accessibility

Accessibility related to the availability and accessibility of the service and was identified as a consistent category across all three questions. For example, one respondent indicated a preference for online services as it would be *“quick to get help”*. Regarding the preference for in-person support, six respondents stated similarly *“if they were available”*. A subcategory relating to logistics was also identified, with participants citing factors such as distance from services, travel, cost of services, and appointment times as important considerations for service preferences. For example, telehealth was endorsed provided *“a service was too far away”*.

#### Connection

Connection with a clinician or service, continuity of care, and confidentiality appeared to inform a respondent’s service preference, though less so than their acceptability of telehealth versus in-person services. For example, respondents indicating a preference for in-person support reasoned it was *“better to build rapport with someone”*, and be *“able to establish a good relationship”*. However, one respondent indicated a preference for online services as *“in a small town, [I] would not like to approach a friend of a friend”*.

#### Trusted service

Respondents indicated a preference for a trusted service, based on their own prior experiences, as well as the competency and reputation of such services. For example, one respondent indicated a preference for a psychologist due to *“their level of understanding, professional expertise, training and based on my past experiences”*. Other respondents indicated a preference to access mental health support via their GP as *“he could then refer me to the relevant person or place”*, and *“it’s a good place to start”*. Though this category was more prominent when respondents were providing a rationale for their preferred service, a number of responses (*n* = 25) indicated the acceptability of an in-person service provided their GP referred them. Likewise, a smaller number of responses (*n* = 6) indicated the acceptability of telehealth support should their GP refer them.

### Factors influencing clinician preferences

Participants were asked about the desirable skills and community knowledge base they seek in their mental health clinicians, with three main categories identified: *clinician, connection*, and *cultural awareness*. Some responses (*n* = 20) indicated there was nothing participants wanted their clinician to know about their community with some (*n* = 35) stating that clinical skills were more important. See Table 4 for frequency and percentages of categories.

**Table 4.** What do rural people seek in a clinician?

Category	Example Codes	Skills (N * = 242*)	Community Knowledge (N * = 200*)
		*n*	*n*
Clinician specific		221	35
Competencies	Assessment, knowledge, clinical skills more important	81	35
Clinician qualities	Experience, qualified, effectiveness	140	–
Connection	Connection, continuity of care, confidential	177	20
Cultural awareness		4	210
Local knowledge	Local knowledge		102
Rural knowledge	Rural knowledge	4	65
Accessibility	Local clinician, limited service, stigma	–	43
Community knowledge not important		-	20

*Note. n* represents number of responses provided.

#### Clinician

The clinician category encompassed two sub-categories relating to competencies and clinician qualities. Competencies related to the desire for clinicians to have a strong knowledge base and ability to assess and deliver a wide variety of treatments. Whereas clinician qualities related to the clinician as a person, including their clinical experience, professionalism, and holding some level of relevant qualification. Over half of respondents indicated the importance of such qualities in a clinician, such as *“personal experience with university qualifications and years of hands-on experience”*.

#### Connection

Majority of respondents wrote of skills and qualities necessary to aid the therapeutic relationship, indicating the importance of connection with their clinician. For example, one respondent stated *“time to listen and talk rather than assessing your risk then moving you on”*. Numerous respondents wrote of the importance of being empathetic, non-judgemental, and genuine: *“someone that really sees you as a person not an illness”*.

#### Cultural awareness

The cultural awareness category included sub-categories relating to local and rural knowledge, as well as accessibility of services. Local knowledge was specific to each individual community, such as *“the social-political- environmental landscape, the factors that impact it – natural disasters, population dynamics”*. Or as another respondent wrote: *“I would like them to know a lot about the community and what disasters have happened, where places or towns are”*. Rural knowledge related more broadly to the social-cultural aspects of rural communities (*“the issues around remotely living in an Indigenous community”*), and also rural people (*“the ‘country’ mentality that can be quite negative”*), with one respondent simply stating they would want their clinician to know their community was *“part of my identity”*. Respondents also wrote of the importance of clinicians being aware of mental health service accessibility barriers unique to rural communities: *“how hard it is to get help”*, and *“access to mental health care is hard work, there is a huge stigma, and limited access to supports for many”*.

### Acceptability of telehealth

Service preferences were further explored with participants by examining if telehealth acceptability varied based on the type of clinician providing the service, including city-based clinicians, rural-trained clinicians or rural-based clinicians. Overall, 211 participants completed both the scale and open-ended items related to this research question. As the purpose of the analysis focused on preference rationale, 30 participants were removed due to indicating no preference for clinician type. A further 29 participants were removed due to indicating no preference for telehealth, regardless of clinician type. In total, 152 participants were included in this analysis.

A one-way repeated measures ANOVA indicated a significant effect of clinician type on telehealth acceptability, *F*(1.59, 239.83) = 64.64, *p* = .00, partial η^2^ = .30. Post-hoc pairwise comparisons indicated that a city-based clinician (*M* = 3.0, *SD* = 1.4) was significantly less preferred than either a rural-trained clinician (*M* = 3.5, *SD* = 1.3) or a rural-based clinician (*M* = 4.2, *SD* = .9), with a rural-based clinician the most preferred telehealth option. Overall, 82% of respondents indicated they would *likely* or *very likely* consider using telehealth with a rural-based clinician, compared to 60% of respondents who would consider a rural-trained clinician and 43% of respondents who would consider a city-based clinician.

An open-ended question asking participants to explain their answer was used to further explore respondents’ preferences for telehealth clinician type. Content analysis identified four categories: *cultural awareness, accessibility* and *clinician*. A small number of responses (*n* = 6) acknowledged the importance of *connection* with their clinician. Frequency and percentages of categories are included in Table 5.

**Table 5.** Clinician type preferences (*N* = 152).

Category	Example Codes	Preference Rationale
		*n*
Cultural awareness		52
Rural knowledge	Rural knowledge	41
Local knowledge	Knowledge of services, local knowledge	11
Accessibility	Availability, travel, local clinician	41
Clinician (competencies)	Clinical skills more important, competence	18
Connection	Connection, in person support	4

*Note. n* represents number of responses provided.

#### Cultural awareness

Similar to previous responses, respondents indicated the importance of clinicians having both local and rural knowledge. For example, one respondent who indicated a preference for a rural-based clinician wrote: *“city based city focused clinicians can only help with generalised issues, issues that relate to isolation, lack of services, distance, finances (seasonal farm etc) can only be fully understood by people who have either experienced it or have sought the knowledge and understand that rural/regional/remote communities have differing and specific issues as well as all the general human issues”*. Another wrote: *“rural based clinicians have a better understanding of rural living and struggles, city clinicians would understand but I’m not confident they would at the same extent”*.

#### Accessibility

Accessibility and availability of mental health services also remained an important rationale for all types of telehealth clinician. Some respondents indicated a preference for city-based clinicians as there are *“more options from the city”*, and *“most clinicians don’t live in regional areas”*, with two respondents stating they would *“take whatever they could get*”. Other respondents indicated a preference for a rural-based clinician as, despite it being a telehealth modality, this may reduce the likelihood of travel and other access barriers: *“(it) helps if it’s close, if I have a problem”*.

#### Clinician

Some respondents, again, indicated the importance of clinical skills and competence. One respondent suggested that *“an experienced clinician should be aware of different locations”*. Whilst another wrote: *“cities have all the education and training”*, suggesting that the way in which this category influenced telehealth clinician preferences (that is either city-based or rural-trained/based) varied among participants.

## Discussion

In order to ensure the acceptability of mental health services in rural communities, a greater understanding of service and clinician preferences is needed. The current study aimed to address this gap in the current literature by exploring the rural and regional Australians’ preferences for different service modalities and clinician types. Results suggested an overall preference for in-person support provided by mental health clinicians. However, when asked about telehealth acceptability directly, the majority of participants indicated a likelihood of using telehealth, provided a rural-based clinician was providing the service. Across all questions, respondents’ perspectives were classified into categories relating to *accessibility* and *connection*. For services, additional emphasis was placed on *trusted services* and for clinicians, categories relating to *competencies*, *qualities*, and *cultural awareness* were important.

### Service preferences

The finding that rural people prefer in-person support is consistent with research regarding the broader Australian population (Amos et al., 2022; Mseke et al., 2023), as well as research suggesting that people outside of major cities have a lower preference for telehealth (March et al., 2018). Telehealth and other online services were identified as available supports; however, few respondents indicated a preference for such. Due to limited service availability in rural areas, telehealth is often a referred support, despite client preferences for in-person services (Fisher et al., 2024). Although the majority of respondents indicated already having access to in-person support, over half of the identified services were provided in a medical setting. Again, this finding is consistent with the literature indicating rural people are more likely to receive mental health support via their GP (Batterham et al., 2020; Dolja-Gore et al., 2014), with available supports often less specialised and shorter in duration than specific mental health services (Matta et al., 2021).

### Accessibility

Respondents frequently cited the availability of a service as an important consideration in service usage. The limited services available, in addition to long waiting times and limited specialised support, have been found to be significant barriers to mental health access in rural and regional communities (Kavanagh et al., 2023). Similarly, the affordability and accessibility of a service have been found to be important resource issues for rural people (Kavanagh et al., 2023). For example, the financial and time cost of accessing an essential service, such as mental health support, can be up to seven times more in rural communities than in urban communities (Manning, 2010). Such barriers were reflected in the current study, with respondents suggesting that regardless of service availability, they also needed to consider the accessibility and logistical requirements of services.

### Connection

The category of connection was consistent throughout survey responses, with respondents emphasising the importance of connecting with their clinician. Though the ability to connect and build rapport with clients is a necessary skillset of all mental health providers regardless of location, it is a particularly valued attribute in rural and remote areas (Procter et al., 2015). Rural people have been found to prefer services that allow for relationship building, such as supportive counselling or the use of community support networks (Boyd et al., 2007; Gunn et al., 2013). Clark et al. (2024) suggested that young rural males placed greater importance on genuine connection than on clinical expertise when engaging in mental health services. As some respondents indicated in the current study, the desire for connection influenced service modality preferences with some reporting it is easier to connect with a clinician in person. These responses were similar to previous research which found rural people preferred face-to-face support due to the ability to form stronger connections in person (Howard et al., 2024).

In addition to connection, respondents spoke of the importance of a trusted service and clinician, including the need for clinicians to be competent and effective. As rural communities primarily access services via their GP (Dolja-Gore et al., 2014), the desire for services to be vouched for, either through experience, reputation, or referrals, could perhaps be a product of the current mental health structure. Alternatively, rural mental health practitioners have identified the importance of the generalist skillset and the need for a wide variety of clinical knowledge when practising in rural areas (Sutherland & Chur-Hansen, 2014). The respondents in the current study may be acknowledging the need for clinicians to adequately address the mental health needs of rural communities.

### Cultural awareness

Though clinical knowledge was valuable for respondents, so too was community and rural knowledge, with some respondents acknowledging a cultural divide between urban and rural locales. Such was the importance placed on rural awareness, the majority of respondents indicated increased acceptability of telehealth provided a rural-based practitioner was delivering the service. Understanding an individual’s local and cultural context and how this may impact a person’s presentation have been found to be necessary skills of rural mental health providers (Sutherland & Chur-Hansen, 2014). For example, rural cancer patients have indicated the need to be acknowledged and treated differently from urban clients, with a specific focus on rural relevant information (Gunn et al., 2013).

Community-specific information is also important for clinicians to possess. In the United States, research has found practitioners not only need to understand the historical, social, and familial networks of a rural area (Riding-Malon & Werth, 2014) but also be mindful of the unique economic drivers of a town and how this may impact community members (Bischoff et al., 2014). More locally, a case study examining a community health service found that local providers were attuned to service shortages and community needs, allowing them to problem-solve solutions as necessary (Fitzpatrick et al., 2017).

### Clinical implications

In Australia, rural communities have fewer access to services and experience poorer mental health outcomes than urban communities (AIHW, 2021; Matta et al., 2021). The use of telehealth and other such online supports have been extended as a viable solution to these service shortages (Bradford et al., 2016). As some respondents indicated in the current study, telehealth is necessary to receive support outside of the community, given the challenges of multiple relationships and lack of anonymity that often exist in the rural context. However, the current findings suggest that although telehealth is acceptable in some circumstances, rural people show a preference for in-person support that is accessible and available to them and mental health-specific. The findings also indicate that rural people seek support that is relevant to their local and rural culture in a way that fosters connection and trust.

Taken together, attempting to address mental health challenges in rural areas via clinicians trained in an urban-centric model of mental health (Jameson et al., 2009) may not be appropriate. Amongst GPs, nurses, and allied health clinicians, the need to practice within the rural context has been well researched and has resulted in the development of various rural competency models (Barker et al., 2021; Campbell et al., 2011; Office of the National Rural Health Commissioner, 2023). Yet, despite mental health being the second biggest health issue for rural Australians (Bishop et al., 2017), no such training or guidelines exist for those practising in mental health. The current study demonstrates that rural specific mental health support is wanted by rural communities. Both future research and health providers should explore how to adapt rural mental health services and clinicians to ensure relevancy to both the client and their rural context. Not all rural communities are the same (Bischoff et al., 2014); therefore, care needs to be taken by clinicians to understand the cultural landscape of the community they are supporting.

### Limitations and directions for future research

There were some limitations when conducting the current study. When examining the acceptability of telehealth based on clinician type, some responses indicated that respondents may have misinterpreted the question. For example, responding that they would prefer a rural-based clinician as they would want in-person support. Though the preference for in-person support was reflected in other items during the study, some respondents highlighted potential barriers to telehealth acceptability not explored in the questionnaire, such as respondents who indicated under no circumstances would they use telehealth. Further research may consider examining the acceptability of telehealth more directly. Attitudes such as stigma, which are commonly associated with lower help-seeking (Cheesmond et al., 2019), were not explored in the current study and may have impacted the way in which participants responded to the questions. Though attitudinal barriers may impact initial help-seeking, external barriers (such as service access) impact accessing adequate and ongoing care (Handley et al., 2014). As the current study focused on broader mental health services, including structural and clinician factors, the impacts of attitudinal barriers were not examined.

The current study indicates that further research regarding rural-specific mental health services and clinicians is warranted. More mental health services are needed in rural communities and research should look to exploring services best suited to the needs and wants of such communities.

## Conclusion

The current study examined service and clinician preferences amongst rural Australians in order to improve service acceptability. Results showed a preference for accessible, specialised, in-person services with clinicians who are competent and encourage connection and trust. Participants reported the need for clinicians to be culturally competent in a rural context, demonstrating an increase in the acceptability of telehealth for clinicians with local and rural knowledge. Services that are available to rural Australians need to be acceptable and relevant in order to best meet mental health needs and ensure the uptake of available services. Future research is encouraged to examine how services and clinicians may best adapt to the service wants and needs of rural Australians.

## Data Availability

The data that support the findings of this study are available from the corresponding author upon reasonable request.
